# Nonparametric mixed exponentially weighted moving average-moving average control chart

**DOI:** 10.1038/s41598-024-57407-1

**Published:** 2024-03-21

**Authors:** Muhammad Ali Raza, Azka Amin, Muhammad Aslam, Tahir Nawaz, Muhammad Irfan, Farah Tariq

**Affiliations:** 1 Department of Statistics, Government College University Faisalabad, Faisalabad, 38000 Pakistan; 2 Department of Statistics, Faculty of Science, King Abdulaziz University, Jeddah, Saudi Arabia

**Keywords:** Control chart, Exponentially weighted moving average statistic, Moving average, Monte Carlo simulation, Nonparametric tests, Engineering, Mathematics and computing

## Abstract

This research designed a distribution-free mixed exponentially weighted moving average-moving average (EWMA-MA) control chart based on signed-rank statistic to effectively identify changes in the process location. The EWMA-MA charting statistic assigns more weight to information obtained from the recent $w$ samples and exponentially decreasing weights to information accumulated from all other past samples. The run-length profile of the proposed chart is obtained by employing Monte Carlo simulation techniques. The effectiveness of the proposed chart is evaluated under symmetrical distributions using a variety of individual and overall performance measures. The analysis of the run-length profile indicates that the proposed chart performs better than the existing control charts discussed in the literature. Additionally, an application from a gas turbine is provided to demonstrate how the proposed chart can be used in practice.

## Introduction

The term statistical process control (SPC) refers to a variety of analytical and statistical methods used to enhance products’ quality. Among the SPC methods, the control chart plays an essential role in overseeing manufacturing processes and identifying the presence of special cause variation(s). The origin of the control charting techniques dated back to the 1920s anticipated by Walter A. Shewhart to identify the occurrence of assignable causes of variability in manufacturing processes^1^. The Shewhart control chart, best known for its simplicity, is unfortunately ineffective in detecting small to moderate changes in a process due to its memory-less nature. Later, researchers introduced several memory-type control charts, such as the cumulative sum (CUSUM)^2^, Exponentially weighted moving average (EWMA)^3^, and Moving average (MA)^4^ control charts. These memory-type control charts have gained popularity among practitioners to detect small to moderate process changes, which results in improved process monitoring.

In the literature, combined or mixed control charts were introduced to improve the overall shift detection ability of the existing control charts for a range of shifts. For instance, a combined Shewhart-CUSUM chart was developed by Lucas^5^ that leverages the strengths of both charts to improve the detection capabilities for both large and small changes in a process. Likewise, Lucas and Saccucci^6^ proposed combining Shewhart-EWMA charts to effectively identify both small and large shifts in the process. Klein^7^ evaluated the composite Shewhart-EWMA control charting schemes for enhancing the detection of smaller shifts in monitoring the process mean. Han et al.^8^ developed the CUSUM and EWMA multi-charts to achieve a comprehensive and robust monitoring system capable of identifying the range of shifts in the process mean. Haq^9^ proposed the hybrid exponentially weighted moving average (HEWMA) chart by merging two EWMA statistics to effectively identify the small shifts in the process. Abbas et al.^10^ introduced the mixed EWMA-CUSUM (MEC) chart, offering an efficient approach to monitor minor changes in the process location. Subsequently, Zaman et al.^11^ developed a reverse MEC called a mixed CUSUM-EWMA (MCE) chart to monitor small to moderate changes in the process location. Khoo and Wong^12^ introduced a double moving average (DMA) control chart by merging two MA statistics that was redesigned by Alevizakos et al.^13^ through the correct specification of variance expression of the DMA statistic for efficient detection of small shifts in the process. Interested readers may see Ajadi and Riaz^14^, Osei-Aning et al.^15^, Adeoti and Malela-Majika^16^, and Alevizakos et al.^17^.

All the control charts discussed above assume the normality of the underlying process distribution (or other known distribution models). In practice, the normality assumption is not always met, which may result in the performance deterioration of traditional control charts^18,19^. In this context, nonparametric control charts provide a robust alternative for effective monitoring of process parameters. Furthermore, nonparametric control charts exhibit consistent in-control run-lengths across different continuous distributions^20^. Nonparametric control charts are developed using a variety of statistical tests, such as the precedence statistic, Mann-Whitney test, sign test, Wilcoxon signed-rank test, Wilcoxon rank-sum test, etc.

In the literature, nonparametric control charts have been recommended to efficiently monitor the location of a process, For instance, Bakir and Reynolds^21^ proposed the non-parametric CUSUM control chart that utilizes Wilcoxon's signed-rank statistic with ranks calculated within groups. Amin and Searcy^22^ introduced the EWMA control chart that utilized the signed-rank statistic to effectively monitor the location parameter of a process. Amin et al.^23^ used sign test statistics in the Shewhart and CUSUM charting structure, which provides an efficient method for identifying changes in the process mean. Bakir^24^ proposed Shewhart-type, EWMA-type, and CUSUM-type control charts using a signed-rank statistic. Li et al.^25^ proposed the idea of utilizing Wilcoxon's signed-rank statistic to develop the CUSUM and EWMA control charts for quicker identification of shifts in the process. Similarly, Yang et al.^26^ introduced a novel method known as the Arcsine EWMA sign control chart to more effectively monitor the process mean, aimed at detecting small to moderate shifts. Malela‐Majika and Rapoo^27^ introduced the new control charts called the combined CUSUM-EWMA control chart and its reverse EWMA-CUSUM control chart, which utilized the Wilcoxon rank-sum statistics. Later on, Raza et al.^28^ introduced the distribution-free double EWMA signed-rank (DEWMA-SR) control chart to effectively detect shifts in the process mean. Mabude et al.^29^ designed the generally weighted moving average (GWMA) control chart by using the Wilcoxon rank-sum statistic for improved detection of shifts in location parameter. Alevizakos et al.^30^ introduced the triple EWMA control chart (TEWMA) based on a signed-rank statistic to detect very small changes in the process mean. Rasheed et al.^31^ designed an enhanced version of the non-parametric TEWMA control chart under ranked set sampling for identifying process location shifts. Petcharat and Sukparungsee^32^ proposed the modified exponentially weighted moving average (MEWMA) control chart by utilizing signed-rank statistics to monitor both moderate and large process mean shifts. Abbas et al.^33^ developed the nonparametric progressive mean control by using the Wilcoxon signed-rank statistic (NPPM-SR) to swiftly identify the shifts in process target. Shafqat et al.^34^ proposed the EWMA-SR and homogeneously weighted moving average signed-rank (HWMA-SR) repetitive control charts for prompt detection of small shifts in the location parameter using auxiliary information. For more details, see Letshedi et al.^35^ Qiao and Han^36^, Hou and Yu^37^.

Recently, Sukparungsee et al.^38^ introduced the mixed exponentially weighted moving average-moving average (EWMA-MA) control chart specifically designed for monitoring the process mean under the normal process. However, they neglected the covariance term in the variance expression by assuming that the moving averages are independent. This presumption caused a significant inaccuracy in the variance computation that was amended by Raza et al.^39^ with the correct specification of variance expression. The robustness analysis found that when the smoothing parameter $\left(\lambda \right)$ is adjusted to smaller values, the control chart demonstrated resilience to non-normality. However, it is observed that the performance of the control chart deteriorates with increasing values of $\lambda .$ To solve this issue, we propose a distribution-free mixed EWMA-MA signed-rank control chart for detecting shifts in the location parameter. The signed-rank statistic used in this study is found to be more efficient and has greater statistical power compared to sign statistic because it considers the observations’ magnitude in addition to the signs (see, Graham et al.^40^ and Hollander et al.^41^). The proposed control chart would be an alternative choice for practitioners and applicable in situations where the process distribution is either unknown or non-normal when quick detection of shift in the process location is of paramount interest. For instance, the proposed methodology can be used to monitor the inside diameters of piston rings manufactured by a forging process considered by Graham et al.^42^, the flow width of resist in hard-bake process used by Alevizakos et al.^43^, the filled liquid volume of soft drink beverage bottles considered by Raza et al.^44,45^. Moreover, to signify the practical implementation of the proposal a gas turbine data is used to monitor the ambient humidity which is an important characteristic that effects the CO and NOx emissions.

The rest of the paper is organized as follows: Sect. "The design structure of the EWMA-MA signed-rank control chart" presents the charting structure of the distribution-free mixed EWMA-MA signed-rank control chart. Section "Performance evaluation" assesses the run-length performance of the proposed chart under various symmetric distributions. In Sect. "Comparative study", a comparative study is carried out to evaluate the performance of the proposal compared to its competitors. To validate the proposed chart's practicability, an application to monitor the ambient humidity generated from the gas turbine is presented in Sect. "Real-life example". Finally, Sect. "Summary and conclusion" concludes the paper.

## The design structure of the EWMA-MA signed-rank control chart

Consider a quality characteristic $\left(X\right)$ with known median $\left(\theta \right)$ as a target value. Let ${X}_{ij}$ be the ${i}^{th}$ observation within the ${j}^{th}$ sample or subgroup of size $n(>1)$, where $i=\mathrm{1,2},\dots ,n$ and $j=\mathrm{1,2},\dots$. Furthermore, ${R}_{ij}^{+}$ is the rank assigned to the absolute differences from the targeted value $\theta$, i.e. $\left|{X}_{ij}-\theta \right|$. The signed-rank statistic $\left({SR}_{j}\right)$ is defined as:

1 $${SR}_{j}=\sum_{i=1}^{n}{I}_{ij}{R}_{ij}^{+},$$

where

$$I_{{ij}}  = \left\{ {\begin{array}{*{20}c}    {1,{\mkern 1mu} {\mkern 1mu} \,\,for\left( {X_{{ij}}  - \theta } \right)} & { > 0}  \\    {0,{\mkern 1mu} \,\,{\mkern 1mu} for\left( {X_{{ij}}  - \theta } \right)} & { = 0}  \\    { - 1,{\mkern 1mu} {\mkern 1mu} \,\,for\left( {X_{{ij}}  - \theta } \right)} & { < 0}  \\   \end{array} } \right., $$

The $SR$ statistic is a linear function of the Mann-Whitney statistic $\left({M}_{n}^{+}\right)$, i.e., $SR=2{M}_{n}^{+}-n(n+1) /2$ (for more details, see Gibbons and Chakraborti^46^). The $SR$ statistic has a zero mean and $n(n+1)(2n+1)/6$ variance. The distribution-free mixed EWMA-MA signed-rank statistic is developed by integrating $MA$ statistic into $EWMA$ statistic. The moving average statistic ${MA}_{j}$ of span $w$ at time $j$ is:

2 $$ MA_{j}  = \left\{ {\begin{array}{*{20}c}    {\frac{{\sum _{{k = 1}}^{j} SR_{k} }}{j},\,\,\,for{\mkern 1mu} {\mkern 1mu} } & {j < w}  \\    {\frac{{\sum _{{k = j - w + 1}}^{j} SR_{k} }}{w},\,\,for{\mkern 1mu} {\mkern 1mu} } & {j \ge w}  \\   \end{array} } \right. $$

The mean of the moving average is $E\left({MA}_{j}\right)={\mu }_{0}=0$. The monitoring statistic of EWMA-MA signed-rank control chart is defined as:

3 $${E}_{{SR}_{j}}=\lambda {MA}_{j}+\left(1-\lambda \right){E}_{{SR}_{j-1} },\,\,\,\, j=1, 2, 3,\dots $$

where $\lambda$ is the smooting constant $\left(0<\lambda <1\right)$. The initial value of ${E}_{{SR}_{j}}$ is taken as the mean of $SR$ statistic, i.e. ${E}_{{SR}_{0}}=E\left(SR\right)={\mu }_{0}=0$.

Now, the statistic ${E}_{{SR}_{j}}$ can also be expanded as:

4 $${E}_{{SR}_{j}}=\uplambda {\sum }_{k=0}^{j-1}{\left(1-\lambda \right)}^{k}{MA}_{j-k}+ {\left(1-\lambda \right)}^{j}{E}_{{SR}_{0}}.$$

The in-control (IC) expected value of the ${E}_{{SR}_{j}}$ is:

5 $$ \begin{aligned} E\left( {E_{{SR_{j} }} } \right) & = \lambda \mathop \sum \limits_{k = 0}^{j - 1} \left( {1 - \lambda } \right)^{k} E(MA_{j - k} ) + \left( {1 - \lambda } \right)^{j} E\left( {E_{{SR_{0} }} } \right) \\ \, & = \lambda \mathop \sum \limits_{k = 0}^{j - 1} \left( {1 - \lambda } \right)^{k} \mu_{0} + \left( {1 - \lambda } \right)^{j} \mu_{0} \\ &= \,0. \\ \end{aligned} $$

To obtain the variance of the statistic ${E}_{{SR}_{j}}$, we apply variance on both sides of Eq. (4) and get:

6 $$ \begin{aligned} Var\left( {E_{{SR_{j} }} } \right) & = \lambda^{2} \mathop \sum \limits_{k = 0}^{j - 1} \left( {1 - \lambda } \right)^{2k} Var\left( {MA_{j - k} } \right) + 2\lambda^{2} \mathop \sum \limits_{{1 \le k_{1} < k_{2} \le j}}^{j - 1} \left( {1 - \lambda } \right)^{{j - k_{1} }} \left( {1 - \lambda } \right)^{{j - k_{2} }} Cov\left( {MA_{{k_{1} }} ,MA_{{k_{2} }} } \right) \\ = \lambda^{2} \mathop \sum \limits_{k = 0}^{j - 1} \left( {1 - \lambda } \right)^{2k} Var\left( {MA_{j - k} } \right) \\ + 2\lambda^{2} \mathop \sum \limits_{{k_{1} = 1}}^{j - 1} \mathop \sum \limits_{{k_{2} = k_{1} + 1}}^{j} \left( {1 - \lambda } \right)^{{2j - k_{1} - k_{2} }} Cov\left( {MA_{{k_{1} }} ,MA_{{k_{2} }} } \right), \\ \end{aligned} $$

where, the variance and covariance of $MA$ statistics are, respectively, given as:

7 $$Var\left({MA}_{j}\right)=\left\{\begin{array}{c}\frac{n\left(n+1\right)\left(2n+1\right)}{6j} ,\quad for\,\,  j<w\\ \frac{n\left(n+1\right)\left(2n+1\right)}{6w} ,\quad for \,\,  j\ge w\end{array}\right.$$

8 $$COV\left( {MA_{{k_{1} }} ,MA_{{k_{2} }} } \right) = \left\{ {\begin{array}{*{20}c}    {\frac{{n\left( {n + 1} \right)\left( {2n + 1} \right)}}{{6k_{2} }}} & {\left( {k_{1} ,k_{2} } \right) < w}  \\    {\frac{{\left( {k_{1}  - k_{2}  + w} \right)}}{{k_{1} w}}\frac{{n\left( {n + 1} \right)\left( {2n + 1} \right)}}{6}} & {k_{1}  \ge w,k_{2}  \ge w,\left( {k_{2}  - k_{1} } \right) < w}  \\    {\frac{{\left( {k_{1}  - k_{2}  + w} \right)}}{{w^{2} }}\frac{{n\left( {n + 1} \right)\left( {2n + 1} \right)}}{6}} & {(k_{1} ,k_{2} ) \ge w,\left( {k_{2}  - k_{1} } \right) < w}  \\    0 & {(k_{1} ,k_{2} ) \ge w,\left( {k_{2}  - k_{1} } \right) \ge w}  \\   \end{array} } \right. $$

The center line $\left(CL\right)$, lower control limit $\left(LCL\right)$, and upper control limit $\left(UCL\right)$ for the EWMA-MA signed-rank control chart are determined as:

9 $$\left.\begin{array}{c}{UCL}_{j}=L\sqrt{Var\left({E}_{{SR}_{j}}\right)} \\ CL=0 \\ {LCL}_{j}=-L\sqrt{Var\left({E}_{{SR}_{j}}\right)}\end{array}\right\},$$

where $L>0$ is the width of the control limits. The monitoring statistics ${E}_{{SR}_{j}}$ are plotted against their respective control limits. If either ${E}_{{SR}_{j}}\ge UCL$ or ${E}_{{SR}_{j}}\le LCL$, then process is considered as out-of-control (OOC). In such a case, it is crucial for a quality practitioner to thoroughly investigate the process and detect the assignable cause(s). On the other hand, if $LCL{<E}_{{SR}_{j}}<UCL$, the process is declared as stable or in-control (IC), indicating that no shift has been detected and the process is operating within acceptable limits. The suggested EWMA-MA control chart encompasses the nonparametric EWMA control chart introduced by Amin and Searcy^22^ when $w=1$ and the MA signed-rank control chart for $\lambda =1$. Knoth et al.^47^ criticized mixed control charts by claiming that these control charts assign more weights to past data values than current ones. Recently, contrary to the findings of Knoth et al.^47^, Alevizakos et al.^48^ evaluated the performance of the various mixed memory type EWMA control charts and showed that these charts have superior OOC zero-state and steady-state run length performance, especially for smaller to moderate shifts. It is to be noted that the EWMA-MA statistic assigns more weight to the current '$w$' observations while exponentially decreasing weights to the rest of the observations. It is due to the reliance of the MA statistic on the current $w$ observations. As a result, the weighting structure EWMA-MA statistic matches with the conventional EWMA for observations older than $w$, i.e. their weight decreases exponentially.

## Performance evaluation

To evaluate the effectiveness of the control chart, the average run-length $$(ARL)$$ is commonly used to quantify the average number of samples displayed on a control chart before the occurrence of the first OOC signal^49^. The IC and OOC average run-length are denoted by $${ARL}_{0}$$ and $${ARL}_{1}$$, respectively. If the process is IC, $${ARL}_{0}$$ is typically set to be sufficiently large to minimize the false alarm. Conversely, the $${ARL}_{1}$$ should be small to quickly identify any process shift. To gain deeper insights into the run-length distribution and evaluate the performance of the chart, additional performance metrics such as the standard deviation of run-length ($$SDRL)$$ and median run-length ($$MRL)$$ are used in the literature^50–53^. The performance metrics discussed earlier are used for specific process shifts. To assess overall performance for a range of shifts, additional metrics like the average extra quadratic loss $$(AEQL)$$ and relative mean index $$(RMI)$$ are computed in this study. The $$AEQL$$ is the weighted average of $$ARL$$ calculated for different shifts considered in a process. More information about $$AEQL$$ may be found in Raza et al.^39^ and Malela-Majika^54^. The algebraic expression of $$AEQL$$ is as follows:

10 $$AEQL=\frac{1}{{\delta }_{max}-{\delta }_{min}}\sum_{\delta =0}^{{\delta }_{max}}{\delta }^{2}ARL\left(\delta \right),$$

where, $\delta$ represents the shift’s magnitude, $ARL\left(\delta \right)$ is a $ARL$ value at a specific shift $\delta$ in a process, ${\delta }_{max}$ and ${\delta }_{min}$ indicate the highest and lowest values of the shifts taken into consideration, respectively. A smaller $AEQL$ value indicates its ability to identify process shifts quickly. Han and Tsung^55^ introduced the $RMI$ which is based on the relative difference of the $ARL$ values. $RMI$ is mathematically defined as:

11 $$RMI=\frac{1}{N}{\sum }_{i=1}^{N}\left\{\frac{ARL\left({(\delta }_{i}\right)-{ARL}^{*}\left({(\delta }_{i}\right)}{{ARL}^{*}\left({(\delta }_{i}\right)}\right\},$$

where $ARL$ $\left({\delta }_{i}\right)$ refers to the $ARL$ value of the control chart under the specified shift, and ${ARL}_{({\delta }_{i})}^{*}$ denotes the smallest $ARL$ value across all the control charts that are considered for the comparison under the shift ${\delta }_{i}$. $N$ represents the total number of shifts considered for comparative purposes. The superiority of the control chart is determined by its lower $RMI$ value when compared to other control charts.

In this research, a Monte Carlo simulation is used as a computational technique to obtain numerical findings for evaluating the performance of the control charts. With the help of R software, 10,000 iterations are used to determine the $ARL$, $SDRL$, and $MRL$ values. To achieve the intended ${ARL}_{0}$, several combinations of the design parameters $(\lambda , w)$ and the limit coefficient $(L)$ are tested during the simulation method. The charting statistics ${SR}_{j}$ is of a discrete nature, so it is not always possible to achieve the exact desired, ${ARL}_{0}.$ Therefore, we endure the 1% of variation in desired ${ARL}_{0}$. The run-length characteristics of the EWMA-MA signed-rank control chart are calculated using the following algorithm:

### Calculating the IC run-length profile

i. Choose a specific distribution, such as the normal distribution with mean ${\mu }_{0}$ and variance ${\sigma }^{2}$ to produce 10,000 random samples of size $n$.
ii. Select suitable values for $\lambda$ and $w$.
iii. To achieve a desired ${ARL}_{0}$, such as $370,$ we must identify the appropriate $L$ value while maintaining $n$, $\lambda$, and $w$ as constants.
iv. Calculate the ${SR}_{j}$ statistic from Eq. (3) and subsequently compute the monitoring statistic ${E}_{{SR}_{j}}$.
v. Compare the monitoring statistic ${E}_{{SR}_{j}}$ with the respective control limits given in Eq. (9).
vi. The number of samples is recorded before the monitoring statistic first exceeds the control limit, which is defined as a run-length.
vii. Steps 1 through 6 are repeated 10,000 times to acquire $ARL$.
viii. If the value of $ARL$ is approximately equal to the desired ${ARL}_{0}$, proceed to compute $SDRL$ and $MRL,$ then move on to the next steps. Otherwise, change the value of $L$ and repeat Steps 1 to 7 until the desired ${ARL}_{0}$ is achieved.

### Calculating the OOC run-length profile

ix. A process shift $(\delta \ne 0)$ is introduced to obtain a test sample of size $n$ to simulate the OOC process state, i.e. generating samples from a normal distribution with a shifted mean ${\mu }_{1}={\mu }_{0}+\delta \sigma$ and variance ${\sigma }^{2}$.
x. To determine the run-length characteristics under the OOC scenario, Steps 4 through 7 are iteratively executed 10,000 times and subsequently the values of ${ARL}_{1},{SDRL}_{1},$ and ${MRL}_{1}$ are obtained based on the OOC run-lengths.
xi. After computing the value of ${ARL}_{1}$ for all shifts examined in the study, the $AEQL$ is calculated as a measure of the overall performance evaluation for the EWMA-MA signed-rank control chart.

The values of the limit coefficient $(L)$ for the EWMA-MA signed-rank control chart were obtained by using the aforementioned algorithm for various combinations of sample size $\left(n\right)$, span $(w)$, and smoothing parameter $(\lambda )$ under the fixed ${ARL}_{0}\cong 370$. The results under various parameter settings are displayed in Table 1 which are summarized as:

i. For a specified value of $n$ and $\lambda$, the value of the limit coefficient $L$ decreases as $w$ increases to achieve the desired ${ARL}_{0}$. For example, if we fix $n=10$ and $\lambda =0.05$, then the value of $L$ is $2.304$ for $w=5$ and it decreases to $2.205$ for $w=10$.
ii. Similarly, if $n$ and $w$ are fixed, the value of the limit coefficient increases with $\lambda$. For instance, with $n=12$ and $w=5$, the values of $L$ are $2.305$ and $2.481$ for $\lambda =0.05$ and $0.10$, respectively.
iii. The value of the limit coefficient changes slightly with sample size $n$ by keeping other design parameters as fixed.

**Table 1:** The limit coefficient $(L)$ values for various combinations of $(n, w, \lambda )$ at ${ARL}_{0}\cong 370$.

$\lambda$	$w$	$n$												
		8	9	10	11	12	13	14	15	16	17	18	19	20
0.05	2	2.419	2.417	2.416	2.420	2.419	2.420	2.419	2.420	2.420	2.421	2.419	2.419	2.420
	3	2.372	2.370	2.372	2.373	2.374	2.373	2.375	2.374	2.375	2.374	2.375	2.373	2.371
	4	2.325	2.336	2.334	2.336	2.336	2.333	2.335	2.336	2.335	2.335	2.335	2.334	2.336
	5	2.301	2.301	2.304	2.303	2.305	2.306	2.308	2.305	2.304	2.305	2.309	2.308	2.308
	8	2.231	2.233	2.233	2.236	2.234	2.237	2.237	2.236	2.238	2.239	2.238	2.238	2.238
	10	2.205	2.204	2.205	2.204	2.202	2.207	2.204	2.201	2.205	2.205	2.204	2.200	2.204
0.10	2	2.602	2.604	2.610	2.610	2.607	2.609	2.606	2.610	2.614	2.610	2.612	2.616	2.615
	3	2.547	2.549	2.554	2.560	2.559	2.560	2.559	2.560	2.561	2.558	2.561	2.560	2.561
	4	2.510	2.511	2.512	2.513	2.515	2.516	2.516	2.515	2.518	2.516	2.517	2.517	2.517
	5	2.480	2.479	2.478	2.477	2.481	2.480	2.290	2.475	2.481	2.481	2.481	2.481	2.481
	8	2.401	2.400	2.402	2.405	2.401	2.402	2.403	2.402	2.404	2.402	2.408	2.408	2.405
	10	2.364	2.364	2.363	2.360	2.365	2.364	2.364	2.366	2.365	2.365	2.366	2.368	2.367
0.25	2	2.760	2.762	2.768	2.769	2.778	2.779	2.780	2.784	2.785	2.788	2.789	2.793	2.793
	3	2.710	2.717	2.722	2.724	2.726	2.728	2.737	2.737	2.736	2.737	2.737	2.739	2.739
	4	2.675	2.678	2.684	2.683	2.685	2.687	2.689	2.693	2.696	2.696	2.694	2.699	2.696
	5	2.641	2.649	2.653	2.655	2.657	2.658	2.660	2.662	2.660	2.661	2.661	2.664	2.660
	8	2.579	2.579	2.581	2.580	2.581	2.580	2.580	2.581	2.581	2.580	2.586	2.588	2.584
	10	2.542	2.543	2.543	2.544	2.540	2.546	2.540	2.547	2.546	2.548	2.545	2.547	2.552

The performance and robustness of the nonparametric EWMA-MA signed-rank control chart were determined by assessing shift detection ability for a range of symmetrical distributions, including the standard normal distribution $N(\mathrm{0,1})$; the Logistic distribution, $LG\left(0,\frac{\sqrt{3}}{\pi }\right)$; the Student’s t distribution, $t\left(4\right)$ and $t(10)$; the Laplace distribution, $Laplace\left(0,\frac{1}{\sqrt{2}}\right)$; as well as the contaminated normal $(CN)$. The $CN$ is defined as the combination of two normal distributions with common mean $\mu$ and different variances, i.e., $\left(1-\beta \right)N\left(\mu ,{\sigma }_{1}^{2}\right)+\beta N\left(\mu ,{\sigma }_{2}^{2}\right)$, where ${\sigma }_{1}=2{\sigma }_{2}$ and proportion of contamination is $\beta =0.10.$ For ${ARL}_{0}\cong 370$, $n=10$, and various combinations of design parameters ($\lambda , w,L$), Tables 2, 3, 4, 5, 6 and 7 display the computed run-length characteristics of the proposal under these distributions. The following observations are made from Tables 2, 3, 4, 5, 6 and 7:

i. The results depicts that the IC run-length distribution of the EWMA-MA signed-rank chart remains the same across the various process distributions considered in this study, which is in line with the distribution-free control charting theory.
ii. The OOC run length performance of the proposed chart to detect smaller shifts improves as the value of $w$ increases under a fixed sample size $n$ and sensitivity parameter $\lambda$. For instance, for $n=10$, $\lambda =0.05$ and specified shift size $\delta =0.05$, the ${ARL}_{1}$ value of the proposed chart decreases to $139.1$ from $143.7$ and ${MRL}_{1}$ decreases to $98$ from $106$ when $w$ increases from $5$ to $10$ under student’s $t$ distribution with $10$ degrees of freedom (cf. Tables 2 and 5). In general, the choice of $w$ depends on the shift size that needs to be detected quickly. If smaller shift is of interest then a large value of $w$ should be taken and conversely, a lower value is beneficial for larger shifts.
iii. The OOC run-lengths tend to increase with $\lambda$ for small to moderate shifts $\left(\delta \le 1.0\right)$ under fixed $n$ and $w$. For example, under the shifted process with $\delta =0.10$, $n=10$, and $w=5$, the ${ARL}_{1}$ increases to 57.1 from 46.4 and ${MRL}_{1}$ increases to $43$ from $37$ when $\lambda$ increases from $0.05$ to $0.10$ under the CN distribution (cf. Tables 2 and 3).

**Table 2:** The run-length profile of the EWMA-MA signed-rank control chart under symmetrical distributions for $\lambda =0.05, w=5,n=10,$ and $L=2.304$ at ${ARL}_{0}\approx 370$.

Distribution	Characteristic	$\delta$										
		0	0.05	0.10	0.25	0.50	0.75	1.00	1.50	2.00	2.5	3.0
$N(\mathrm{0,1})$	${\text{ARL}}$	372.5	141.6	50.7	12.5	4.6	2.5	1.6	1.1	1	1	1
	${\text{SDRL}}$	364.1	129.1	42.8	7.7	2.7	1.5	0.8	0.3	0.1	0	0
	${\text{MRL}}$	262	103	39	11	4	2	1	1	1	1	1
$t(4)$	${\text{ARL}}$	372.5	142.2	36.6	9.3	3.5	2	1.4	1.1	1	1	1
	${\text{SDRL}}$	369.8	127.8	28.2	5.6	2.2	1.2	0.7	0.3	0.2	0.1	0.1
	${\text{MRL}}$	258	103	30	9	3	2	1	1	1	1	1
$t(10)$	${\text{ARL}}$	371.8	143.7	46	11.5	4.3	2.3	1.6	1.1	1	1	1
	${\text{SDRL}}$	370.1	128.9	37.9	7.1	2.6	1.4	0.8	0.3	0.1	0	0
	${\text{MRL}}$	257	106	36	11	4	2	1	1	1	1	1
$LG\left(0,\frac{\sqrt{3}}{\pi }\right)$	${\text{ARL}}$	373.7	128.2	45.5	11.2	4.1	2.3	1.6	1.1	1	1	1
	${\text{SDRL}}$	365	115.6	37	6.8	2.5	1.4	0.8	0.3	0.1	0.1	0
	${\text{MRL}}$	260	92	36	10	4	2	1	1	1	1	1
$Laplace\left(0,\frac{1}{\sqrt{2}}\right)$	${\text{ARL}}$	375.6	99.9	33.8	8.8	3.5	2.1	1.5	1.1	1.0	1.0	1.0
	${\text{SDRL}}$	375.1	86.1	25.7	5.3	2.2	1.2	0.8	0.4	0.2	0.1	0.0
	${\text{MRL}}$	258	75	28	8	3	2	1	1	1	1	1
$CN$	${\text{ARL}}$	377.2	131.5	46.4	11.4	4.2	2.3	1.5	1.1	1.0	1.0	1.0
	${\text{SDRL}}$	377.8	118.3	38.0	7.0	2.5	1.3	0.8	0.3	0.1	0.0	0.0
	${\text{MRL}}$	261	96	37	10	4	2	1	1	1	1	1

**Table 3:** The run-length profile of the EWMA-MA signed-rank control chart under symmetrical distributions for $\lambda =0.10, w=5,n=10,$ and $L=2.478$ at ${ARL}_{0}\approx 370$.

Distribution	Characteristic	$\updelta$										
		0	0.05	0.10	0.25	0.50	0.75	1.00	1.50	2.00	2.5	3.0
${\text{N}}(\mathrm{0,1})$	${\text{ARL}}$	369.7	168.7	63.7	13.4	4.9	2.7	1.8	1.1	1	1	1
	${\text{SDRL}}$	369.4	163.8	56.3	8.4	2.7	1.5	0.9	0.4	0.1	0	0
	${\text{MRL}}$	258	120	47	12	5	2	2	1	1	1	1
${\text{t}}(4)$	${\text{ARL}}$	377.6	168.6	44.9	9.8	3.8	2.2	1.6	1.1	1	1	1
	${\text{SDRL}}$	378.6	164	38.6	5.8	2.2	1.3	0.8	0.4	0.2	0.1	0.1
	${\text{MRL}}$	264	118	34	9	3	2	1	1	1	1	1
${\text{t}}(10)$	${\text{ARL}}$	380.2	168.4	57	12.4	4.6	2.5	1.7	1.1	1	1	1
	${\text{SDRL}}$	376	165.8	51.2	7.6	2.5	1.5	0.9	0.4	0.2	0.1	0
	${\text{MRL}}$	266	118	42	11	4	2	1	1	1	1	1
${\text{LG}}\left(0,\frac{\sqrt{3}}{\uppi }\right)$	${\text{ARL}}$	366.9	154	55.8	11.9	4.5	2.5	1.7	1.2	1	1	1
	${\text{SDRL}}$	366.7	148.8	49.5	7.1	2.5	1.4	0.9	0.4	0.2	0.1	0
	${\text{MRL}}$	256	107	41	11	4	2	1	1	1	1	1
${\text{Laplace}}\left(0,\frac{1}{\sqrt{2}}\right)$	${\text{ARL}}$	371.4	120.9	40	9.4	3.8	2.3	1.6	1.2	1	1	1
	${\text{SDRL}}$	373.4	116.3	33.3	5.4	2.2	1.3	0.9	0.4	0.2	0.1	0
	${\text{MRL}}$	256	86	31	9	4	2	1	1	1	1	1
${\text{CN}}$	${\text{ARL}}$	372	159	57.1	12.3	4.5	2.5	1.7	1.1	1	1	1
	${\text{SDRL}}$	375.4	155.1	50.2	7.4	2.5	1.4	0.8	0.3	0.1	0	0
	${\text{MRL}}$	253	111	43	11	4	2	1	1	1	1	1

**Table 4:** The run-length profile of the EWMA-MA signed-rank control chart under symmetrical distributions for $\lambda =0.25, w=5,n=10,$ and $L=2.653$ at ${ARL}_{0}\approx 370$.

Distribution	Characteristic	$\updelta$										
		0	0.05	0.10	0.25	0.50	0.75	1.00	1.50	2.00	2.5	3.0
${\text{N}}(\mathrm{0,1})$	${\text{ARL}}$	371	214.4	93.5	15.8	5.1	2.9	2	1.3	1.1	1	1
	${\text{SDRL}}$	379.9	220.8	88.6	11.9	2.5	1.4	0.9	0.5	0.3	0.1	0
	${\text{MRL}}$	256	147	67	12	5	3	2	1	1	1	1
${\text{t}}(4)$	${\text{ARL}}$	368.5	211.5	65.2	11	4	2.4	1.8	1.3	1.1	1	1
	${\text{SDRL}}$	378.3	214.6	62	7.2	2	1.2	0.8	0.5	0.3	0.2	0.1
	${\text{MRL}}$	252	145	47	9	4	2	2	1	1	1	1
${\text{t}}(10)$	${\text{ARL}}$	375.6	211.9	82.6	14.3	4.8	2.8	1.9	1.3	1.1	1	1
	${\text{SDRL}}$	381.3	218.2	80	10.3	2.4	1.3	0.9	0.5	0.3	0.1	0.1
	${\text{MRL}}$	257	143	58	11	5	3	2	1	1	1	1
${\text{LG}}\left(0,\frac{\sqrt{3}}{\uppi }\right)$	${\text{ARL}}$	375.1	197.6	80.8	13.6	4.6	2.7	1.9	1.3	1.1	1	1
	${\text{SDRL}}$	393.2	201.4	76.2	9.8	2.3	1.3	0.9	0.5	0.3	0.2	0.1
	${\text{MRL}}$	259	137	57	11	4	2	2	1	1	1	1
${\text{Laplace}}\left(0,\frac{1}{\sqrt{2}}\right)$	${\text{ARL}}$	373.9	160.7	57.6	10.2	4.0	2.5	1.8	1.3	1.1	1.0	1.0
	${\text{SDRL}}$	384.2	167.1	53.4	6.6	2.0	1.3	0.9	0.5	0.3	0.2	0.1
	${\text{MRL}}$	253	109	41	9	4	2	2	1	1	1	1
${\text{CN}}$	${\text{ARL}}$	377.7	204.8	83.3	14.3	4.8	2.7	1.9	1.3	1.1	1	1
	${\text{SDRL}}$	393.4	210.4	79.2	10.5	2.4	1.3	0.8	0.5	0.2	0.1	0
	${\text{MRL}}$	259	140	59	11	5	2	2	1	1	1	1

**Table 5:** The run-length profile of the EWMA-MA signed-rank control chart under symmetrical distributions for $\lambda =0.05, w=10,n=10,$ and $L=2.205$ at ${ARL}_{0}\approx 370$.

Distribution	Characteristic	$\updelta$										
		0	0.05	0.10	0.25	0.50	0.75	1.00	1.50	2.00	2.5	3.0
${\text{N}}(\mathrm{0,1})$	${\text{ARL}}$	374.1	138.6	51.8	13.3	4.6	2.3	1.5	1.1	1	1	1
	${\text{SDRL}}$	382.6	131.6	42.2	8.4	3.2	1.5	0.8	0.3	0.1	0	0
	${\text{MRL}}$	255	101	41	13	4	2	1	1	1	1	1
${\text{t}}(4)$	${\text{ARL}}$	375.7	138.8	38.6	10.2	3.4	1.9	1.4	1.1	1	1	1
	${\text{SDRL}}$	382.9	131.5	29.2	6.4	2.4	1.1	0.6	0.3	0.2	0.1	0.1
	${\text{MRL}}$	260	99	32	10	3	2	1	1	1	1	1
${\text{t}}(10)$	${\text{ARL}}$	379.5	139.1	47.9	12.5	4.2	2.2	1.5	1.1	1	1	1
	${\text{SDRL}}$	391.6	132.8	38.2	7.7	2.9	1.4	0.7	0.3	0.1	0	0
	${\text{MRL}}$	258	98	39	12	3	2	1	1	1	1	1
${\text{LG}}\left(0,\frac{\sqrt{3}}{\uppi }\right)$	${\text{ARL}}$	380.2	125.5	46	11.9	4.1	2.1	1.5	1.1	1	1	1
	${\text{SDRL}}$	387.6	117	36.6	7.5	2.9	1.3	0.7	0.3	0.1	0.1	0
	${\text{MRL}}$	263	90	37	12	3	2	1	1	1	1	1
${\text{Laplace}}\left(0,\frac{1}{\sqrt{2}}\right)$	${\text{ARL}}$	371.3	97.1	34.7	9.6	3.4	2.0	1.4	1.1	1.0	1.0	1.0
	${\text{SDRL}}$	370.6	87.1	25.9	6.2	2.4	1.2	0.7	0.3	0.2	0.1	0.0
	${\text{MRL}}$	254	72	28	9	3	2	1	1	1	1	1
${\text{CN}}$	${\text{ARL}}$	375.2	130.5	47.3	12.3	4.2	2.1	1.4	1.1	1	1	1
	${\text{SDRL}}$	383.9	122.6	37.9	7.6	2.9	1.3	0.7	0.2	0	0	0
	${\text{MRL}}$	256	94	38	12	3	2	1	1	1	1	1

**Table 6:** The run-length profile of the EWMA-MA signed-rank control chart under symmetrical distributions for $\lambda =0.10, w=10,n=10,$ and $L=2.365$ at ${ARL}_{0}\approx 370$.

Distribution	Characteristic	$\delta$										
		0	0.05	0.10	0.25	0.50	0.75	1.00	1.50	2.00	2.5	3.0
$N(\mathrm{0,1})$	${\text{ARL}}$	367.3	161.7	62.8	13.9	4.9	2.5	1.6	1.1	1	1	1
	${\text{SDRL}}$	386.2	164.5	54.7	8.6	3.1	1.5	0.9	0.3	0.1	0	0
	${\text{MRL}}$	243	111	46	13	4	2	1	1	1	1	1
$t(4)$	${\text{ARL}}$	367.3	161.3	43.5	10.4	3.7	2	1.5	1.1	1	1	1
	${\text{SDRL}}$	384.6	163	36.4	6.1	2.4	1.2	0.7	0.3	0.2	0.1	0.1
	${\text{MRL}}$	247	111	33	10	3	2	1	1	1	1	1
$t(10)$	${\text{ARL}}$	374.2	160.3	57.1	12.7	4.6	2.4	1.6	1.1	1	1	1
	${\text{SDRL}}$	386.1	165.6	49.5	7.6	2.9	1.4	0.8	0.3	0.1	0.1	0
	${\text{MRL}}$	255	109	43	12	4	2	1	1	1	1	1
$LG\left(0,\frac{\sqrt{3}}{\pi }\right)$	${\text{ARL}}$	374.8	147.6	54.4	12.3	4.4	2.3	1.6	1.1	1	1	1
	${\text{SDRL}}$	394.9	149.1	47.1	7.3	2.8	1.4	0.8	0.3	0.1	0.1	0
	${\text{MRL}}$	254	100	40	12	4	2	1	1	1	1	1
$Laplace\left(0,\frac{1}{\sqrt{2}}\right)$	${\text{ARL}}$	375.5	112.5	39.7	9.8	3.7	2.1	1.5	1.1	1.0	1.0	1.0
	${\text{SDRL}}$	395.9	113.4	32.0	5.9	2.4	1.3	0.8	0.4	0.2	0.1	0.0
	${\text{MRL}}$	254	78	31	10	3	2	1	1	1	1	1
$CN$	${\text{ARL}}$	375	151	56.4	12.7	4.4	2.3	1.6	1.1	1	1	1
	${\text{SDRL}}$	392.2	154.7	49.2	7.6	2.8	1.4	0.8	0.3	0.1	0	0
	${\text{MRL}}$	250	103	42	12	4	2	1	1	1	1	1

**Table 7:** The run-length profile of the EWMA-MA signed-rank control chart under symmetrical distributions for $\lambda =0.25, w=10,n=10,$ and $L=2.543$ at ${ARL}_{0}\approx 370$.

Distribution	Characteristic	$\delta$										
		0	0.05	0.10	0.25	0.50	0.75	1.00	1.50	2.00	2.5	3.0
$N(\mathrm{0,1})$	${\text{ARL}}$	370.3	191.2	83.9	14.9	4.9	2.7	1.9	1.2	1	1	1
	${\text{SDRL}}$	408.6	212.2	78.8	10.4	2.8	1.4	0.8	0.4	0.2	0.1	0
	${\text{MRL}}$	246	124	61	13	5	2	2	1	1	1	1
$t(4)$	${\text{ARL}}$	372.3	192.3	57.2	10.7	3.8	2.2	1.6	1.2	1.1	1	1
	${\text{SDRL}}$	418.3	214.4	52.8	6.6	2.1	1.1	0.8	0.4	0.3	0.2	0.1
	${\text{MRL}}$	238	125	41	10	3	2	1	1	1	1	1
$t(10)$	${\text{ARL}}$	376.9	190.5	74.8	13.7	4.6	2.6	1.8	1.2	1.1	1	1
	${\text{SDRL}}$	425.9	210.9	69.8	9.3	2.6	1.3	0.8	0.4	0.2	0.1	0.1
	${\text{MRL}}$	242	124	54	12	4	2	2	1	1	1	1
$LG\left(0,\frac{\sqrt{3}}{\pi }\right)$	${\text{ARL}}$	369.4	174.4	72.2	13.1	4.5	2.5	1.8	1.2	1.1	1	1
	${\text{SDRL}}$	411.8	196.4	66.8	8.4	2.5	1.3	0.8	0.4	0.2	0.1	0.1
	${\text{MRL}}$	235	109	52	12	4	2	2	1	1	1	1
$Laplace\left(0,\frac{1}{\sqrt{2}}\right)$	${\text{ARL}}$	370.7	137.7	50.4	10.1	3.8	2.3	1.7	1.2	1.1	1.0	1.0
	${\text{SDRL}}$	412.3	153.2	45.8	6.2	2.2	1.2	0.8	0.5	0.3	0.1	0.1
	${\text{MRL}}$	236	89	37	9	3	2	2	1	1	1	1
$CN$	${\text{ARL}}$	370	179.8	75	13.4	4.5	2.6	1.8	1.2	1	1	1
	${\text{SDRL}}$	419.1	199.7	69.2	8.9	2.5	1.3	0.8	0.4	0.2	0.1	0
	${\text{MRL}}$	234	116	55	12	4	2	2	1	1	1	1

These findings suggest that a small value of $\lambda$ and a large value of $w$ should be taken if the quick detection of smaller shifts is of primary interest and vice-versa.

## Comparative study

The performance of the EWMA-MA signed-rank control chart is evaluated and compared with other competing control charts like MA sign (MA-SN) and MA signed rank (MA-SR) by Pawar et al.^56^, EWMA sign (EWMA-SN) by Yang et al.^26^, EWMA signed-rank (EWMA-SR) by Graham et al.^42^, and mixed EWMA-CUSUM sign (MEC-SN) by Abbasi et al.^57^. The comparison of the OOC run-length distribution is made under various symmetrical distributions based on different performance metrics such as ${ARL}_{1}$,${SDRL}_{1}$, and ${MRL}_{1}$ for a range of shifts $(\delta )$ in the process. Moreover, the $AEQL$ and $RMI$ are used to assess the overall effectiveness of the proposed control chart in comparison to its competitors.

For a rational comparison between the EWMA-MA signed-rank and existing control charts, the IC run-length is fixed at ${ARL}_{0}=370$ with a sample size $n=10$. The MA-SN and MA-SR control charts were constructed by setting $w=5$, with $k=3.10$ and $2.849$, respectively. Likewise, $\lambda =0.05$ with $k=2.675$ and $2.481$ were used to set up the EWMA-SN and EWMA-SR control charts, respectively. The MEC-SN control chart was computed using the design parameters $\lambda =0.05$,$k=0.5$, and $h=51.28$. Furthermore, the EWMA-MA signed rank control chart was calculated using the parameter settings $w=5$, $\lambda =0.05$, and $L=2.304$. The $ARL$ and $SDRL$ values of each control chart are given in the first row of Table 8, while $MRL$ is provided in the second row. The minimum values of ${ARL}_{1}$,$AEQL$, and $RMI$ are indicated by bold fonts. The following observations are made from Table 8:

i. As the magnitude of the shift increases, the run-length properties associated with OOC conditions exhibit a rapid decrease.
ii. The EWMA-MA signed-rank control chart outperforms its counterparts in detecting a specific shift in the process mean, regardless of distribution type.
iii. The proposed chart exhibits superior overall effectiveness in detecting a range of shifts with smaller values of $AEQL$ and $RMI$ as compared to the existing control charts.

**Table 8:** The run-length characteristics (the first row contain ${ARL}_{1}$ s with ${SDRL}_{1}$ s in parenthesis, while ${MRL}_{1}$ s are in second row) of the existing MA-SN, EWMA-SN, MA-SR, EWMA-SR, MEC-SN, and proposed EWMA-MA(SR) control charts for $n=10$ at ${ARL}_{0}\approx 370$.

Control chart	$\delta$										$AEQL$	$RMI$
	0.10	0.20	0.30	0.40	0.50	0.75	1.00	1.50	2.00	3.0		
	Normal distribution, i.e. $N (\mathrm{0,1})$											
MA-SN with $w=5, k=3.10$	173.1 (168.3) 122	57.3 (56.2) 40	23.5 (21.6) 17	12.0 (10.1) 9	7.3 (5.4) 6	3.4 (1.8) 3	2.3 (1.0) 2	1.5 (0.6) 2	1.2 (0.4) 1	**1.0** (0.1) 1	10.8	0.91
MA-SR with $w=5, k=2.849$	140.9 (137.0) 100	39.6 (37.6) 28	15.7 (13.6) 11	8.2 (6.1) 6	5.3 (3.2) 4	2.9 (1.0) 3	2.2 (0.5) 2	2.0 (0.1) 2	2.0 (0.0) 2	2.0 (0.0) 2	14.3	0.75
EWMA-SN with $\lambda =0.05, k=2.675$	74.2 (59.7) 57	26.7 (15.3) 23	15.3 (7.0) 14	10.7 (4.2) 10	8.4 (2.8) 8	5.4 (1.5) 5	4.1 (1.0) 4	2.9 (0.6) 3	2.4 (0.5) 2	2.0 (0.1) 2	16.6	0.99
EWMA-SR with $\lambda =0.05 , k=2.481$	56.1 (42.4) 44	20.8 (10.6) 18	12.5 (4.8) 11	9.1 (2.8) 9	7.2 (1.9) 7	5.1 (0.9) 5	4.3 (0.5) 4	3.9 (0.3) 4	3.5 (0.5) 3	3.0 (0.2) 3	21.6	1.14
MEC-SN with $\lambda =0.05$, $k=0.5$,$h=51.28$	74.3 (38.5) 64	37.7 (11.6) 35	27.2 (6.0) 26	21.8 (3.9) 21	18.5 (2.9) 18	13.9 (1.8) 14	11.4 (1.3) 11	8.9 (0.8) 9	7.8 (0.6) 8	7.1 (0.3) 7	50.7	3.97
EWMA-MA (SR) with $L=2.304,$ $w=5,\lambda =0.05$	**50.4** (41.4) 40	**17.4** (11.6) 15	**9.6** (5.7) 9	**6.3** (3.7) 6	**4.5** (2.7) 4	**2.5** (1.5) 2	**1.6** (0.8) 1	**1.1** (0.3) 1	**1.0** (0.1) 1	**1.0** (0.0) 1	**7.8**	**0.0**
Student’s t-distribution with $df=5$												
MA-SN with $w=5, k=3.10$	131.0 (127.9) 93	37.2 (35.2) 27	14.9 (13.0) 11	7.8 (5.9) 6	5.0 (3.3) 4	2.7 (1.2) 3	2.0 (0.8) 2	1.4 (0.5) 1	1.2 (0.4) 1	1.1 (0.2) 1	9.6	0.67
MA-SR with $w=5, k=2.849$	113.7 (110.3) 80	28.5 (26.0) 20	11.3 (9.0) 9	6.3 (4.2) 5	4.3 (2.3) 4	2.6 (0.8) 2	2.2 (0.4) 2	2.0 (0.1) 2	2.0 (0.0) 2	2.0 (0.0) 2	13.6	0.71
EWMA-SN with $\lambda =0.05, k=2.675$	54.3 (40.2) 43	20.1 (10.3) 18	12.1 (4.9) 11	8.6 (3.0) 8	6.8 (2.1) 7	4.6 (1.2) 5	3.6 (0.9) 3	2.8 (0.6) 3	2.4 (0.5) 2	2.1 (0.3) 2	16.0	0.93
EWMA-SR with $\lambda =0.05 , k=2.481$	45.9 (31.6) 37	17.6 (8.2) 16	10.8 (3.9) 10	7.9 (2.2) 8	6.5 (1.6) 6	4.8 (0.8) 5	4.2 (0.4) 4	3.8 (0.4) 4	3.5 (0.5) 4	3.2 (0.4) 3	21.8	1.22
MEC-SN with $\lambda =0.05$, $k=0.5$,$h=51.28$	59.9 (26.8) 54	32.0 (8.2) 31	23.4 (4.5) 23	18.9 (3.0) 19	16.2 (2.3) 16	12.4 (1.5) 12	10.5 (1.1) 10	8.6 (0.7) 9	7.9 (0.6) 8	7.3 (0.5) 7	50.1	4.04
EWMA-MA (SR) with $L=2.304, w=5,\lambda =0.05$	**40.6** (32.2) 33	**14.0** (8.8) 13	**8.0** (4.7) 8	**5.3** (3.1) 5	**3.8** (2.3) 3	**2.1** (1.3) 2	**1.5** (0.8) 1	**1.1** (0.3) 1	**1.0** (0.2) 1	**1.0** (0.1) 1	**7.46**	**0.0**
Laplace distribution, i.e.$Laplace\left(0,\frac{1}{\sqrt{2}}\right)$												
MA-SN with $w=5, k=3.10$	81.4 (79.6) 57	21.0 (18.9) 15	9.4 (7.6) 7	5.5 (3.8) 4	4.0 (2.4) 3	2.5 (1.1) 2	2.0 (0.8) 2	1.5 (0.6) 1	1.3 (0.4) 1	1.1 (0.2) 1	9.1	0.40
MA-SR with $w=5, k=2.849$	83.1 (79.6) 59	21.1 (18.8) 15	9.0 (6.9) 7	5.4 (3.4) 4	3.9 (2.0) 3	2.6 (0.8) 2	2.2 (0.4) 2	2.0 (0.1) 2	2.0 (0.0) 2	2.0 (0.0) 2	13.3	0.61
EWMA-SN with $\lambda =0.05, k=2.675$	34.8 (22.2) 29	14.5 (6.4) 13	9.4 (3.4) 9	7.2 (2.3) 7	5.9 (1.7) 6	4.3 (1.1) 4	3.5 (0.8) 3	2.8 (0.6) 3	2.5 (0.5) 2	2.1 (0.3) 2	15.7	0.81
EWMA-SR with $\lambda =0.05 , k=2.481$	37.4 (24.5) 31	15.3 (6.7) 14	9.8 (3.3) 9	7.5 (2.1) 7	6.2 (1.5) 6	4.7 (0.8) 5	4.2 (0.5) 4	3.8 (0.4) 4	3.6 (0.5) 4	3.2 (0.4) 3	21.8	1.21
MEC-SN with $\lambda =0.05$, $k=0.5$,$h=51.28$	44.8 (16.0) 42	26.2 (5.6) 25	20.0 (3.4) 20	16.8 (2.4) 17	14.8 (2.0) 15	11.9 (1.4) 12	10.4 (1.1) 10	8.8 (0.8) 9	8.0 (0.6) 8	7.4 (0.5) 7	50.1	3.94
EWMA-MA (SR) with $L=2.304, w=5,\lambda =0.05$	**33.6** (25.9) 28	**12.2** (7.6) 11	**6.9** (4.1) 7	**4.7** (2.9) 4	**3.5** (2.2) 3	**2.1** (1.2) 2	**1.5** (0.8) 1	**1.1** (0.4) 1	**1.1** (0.2) 1	**1.0** (0.0) 1	**7.32**	**0.0**
Logistic distribution, i.e.$LG\left(0,\frac{\sqrt{3}}{\pi }\right)$												
MA-SN with $w=5, k=3.10$	146.5 (144.0) 103	44.4 (42.6) 31	17.4 (15.5) 13	9.1 (7.2) 7	5.8 (4.1) 5	3.0 (1.4) 3	2.1 (0.8) 2	1.5 (0.6) 1	1.2 (0.4) 1	**1.0** (0.2) 1	9.9	0.73
MA-SR with $w=5, k=2.849$	112.4 (110.7) 79	31.3 (28.7) 23	12.6 (10.3) 10	6.8 (4.7) 5	4.6 (2.7) 4	2.7 (0.9) 2	2.2 (0.5) 2	2.0 (0.1) 2	2.0 (0.0) 2	2.0 (0.0) 2	13.8	0.66
EWMA-SN with $\lambda =0.05, k=2.675$	61.5 (47.2) 48	22.2 (11.8) 20	13.2 (5.6) 12	9.3 (3.3) 9	7.4 (2.4) 7	4.9 (1.3) 5	3.8 (0.9) 4	2.8 (0.6) 3	2.4 (0.5) 2	2.1 (0.3) 2	16.3	0.93
EWMA-SR with $\lambda =0.05 , k=2.481$	50.2 (36.1) 41	18.9 (9.1) 17	11.5 (4.2) 11	8.4 (2.5) 8	6.8 (1.7) 7	5.0 (0.9) 5	4.2 (0.5) 4	3.9 (0.4) 4	3.5 (0.5) 4	3.1 (0.3) 3	21.7	1.17
MEC-SN with $\lambda =0.05$, $k=0.5$,$h=51.28$	65.8 (31.6) 58	34.0 (9.4) 32	24.6 (5.0) 24	20.0 (3.3) 20	17.0 (2.6) 17	13.0 (1.6) 13	10.9 (1.2) 11	8.8 (0.8) 9	7.9 (0.6) 8	7.2 (0.4) 7	50.4	3.96
EWMA-MA (SR) with $L=2.304,$ $w=5,\lambda =0.05$	**44.9** (36.2) 36	**15.5** (10.1) 14	**8.6** (5.0) 8	**5.7** (3.4) 6	**4.2** (2.5) 4	**2.3** (1.4) 2	**1.6** (0.8) 1	**1.1** (0.3) 1	**1.0** (0.1) 1	**1.0** (0.0) 1	**7.65**	**0.0**
Contaminated Normal distribution with 10% contamination proportion												
MA-SN with $w=5, k=3.10$	181.0 (177.4) 128	62.7 (60.5) 44	26.8 (24.7) 19	13.7 (11.7) 10	8.3 (6.6) 6	3.7 (2.1) 3	2.5 (1.1) 2	1.7 (0.6) 2	1.3 (0.5) 1	1.1 (0.3) 1	11.9	0.90
MA-SR with $w=5, k=2.849$	139.4 (134.0) 100	41.4 (39.2) 29	17.6 (15.2) 13	9.2 (7.0) 7	5.9 (3.7) 5	3.2 (1.3) 3	2.4 (0.6) 2	2.0 (0.2) 2	2.0 (0.0) 2	2.0 (0.0) 2	14.6	0.68
EWMA-SN with $\lambda =0.05, k=2.675$	80.0 (65.1) 61	28.9 (17.4) 25	16.4 (7.7) 15	11.6 (4.6) 11	8.9 (3.1) 8	5.7 (1.7) 6	4.3 (1.1) 4	3.1 (0.7) 3	2.6 (0.6) 3	2.1 (0.4) 2	17.7	0.94
EWMA-SR with $\lambda =0.05 , k=2.481$	62.0 (46.6) 49	22.9 (12.0) 20	13.7 (5.6) 12	9.8 (3.2) 9	7.8 (2.2) 7	5.4 (1.1) 5	4.5 (0.6) 4	3.9 (0.3) 4	3.7 (0.5) 4	3.2 (0.4) 3	22.8	1.09
MEC-SN with $\lambda =0.05$, $k=0.5$,$h=51.28$	78.0 (41.9) 67	39.4 (12.4) 37	28.2 (6.5) 27	22.7 (4.2) 22	19.3 (3.2) 19	14.5 (1.9) 14	11.9 (1.4) 12	9.3 (0.9) 9	8.2 (0.6) 8	7.4 (0.5) 7	53.0	3.79
EWMA-MA (SR) with $L=2.304,$ $w=5,\lambda =0.05$	**56.7** (48.5) 44	**19.0** (12.7) 17	**10.7** (6.5) 10	**7.0** (4.1) 7	**5.1** (3.0) 5	**2.8** (1.7) 2	**1.9** (1.0) 2	**1.2** (0.4) 1	**1.0** (0.2) 1	**1.0** (0.1) 1	**8.23**	**0.0**

Significant values are in bold.

## Real-life example

**Figure 1:**
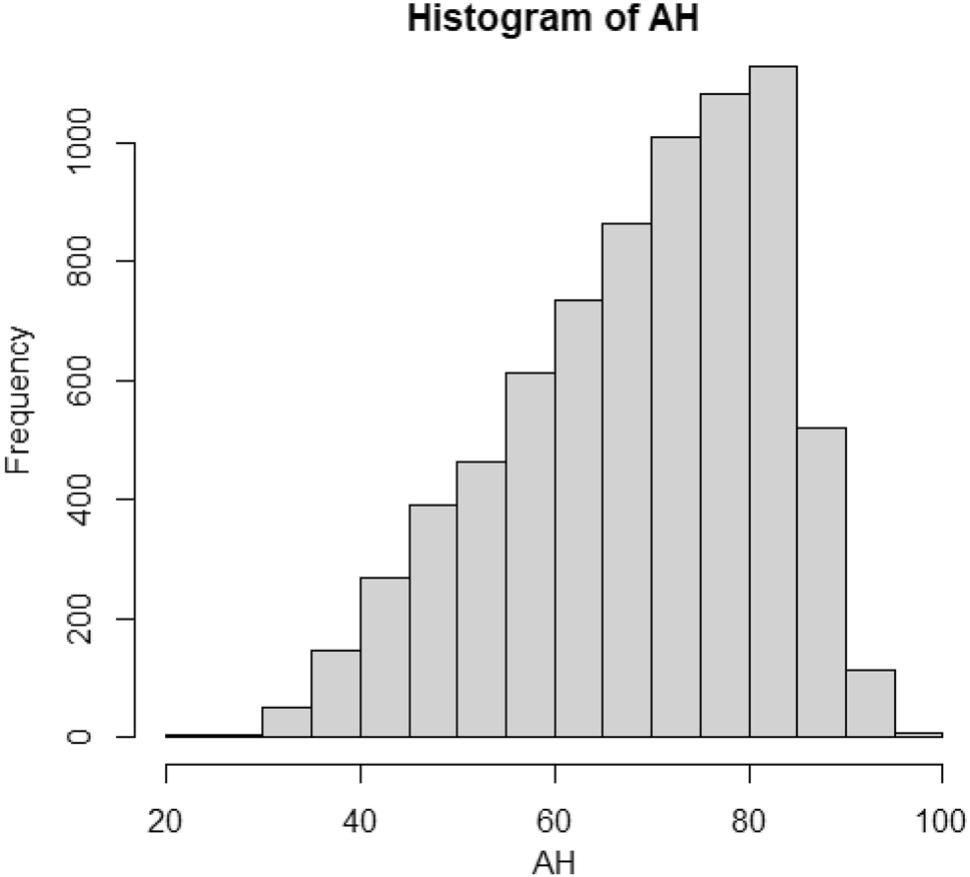
Histogram of AH data.

To demonstrate the applicability and relevance of the EWMA-SR singed-rank chart to real-life scenarios, an industrial dataset of a gas-turbine located in Türkiye^58^ was taken. The dataset consists of 36733 observations covering the period 2011 to 2016 from 11 sensors at hourly intervals. The dataset includes the following main parameters: ambient temperature (AT), ambient humidity (AH), ambient pressure (AP), gas turbine exhaust pressure, air filter differential pressure, turbine inlet temperature, turbine after temperature, turbine energy yield (TEY), carbon monoxide (CO) emissions, compressor discharge pressure, and nitrogen oxide (NOx) emissions. Many researchers used different key factors of combined cycle power plants in their studies to monitor the energy output of the plant. For example, Nawaz and Han^59^ examined the AP as a variable of interest and its impact on the overall performance of the power plant. Similarly, Raza et al.^39^ utilized the AT as a variable of interest to demonstrate how it affects the overall performance of a power plant. In this study, ambient humidity (AH) is selected as a variable of interest that can significantly affect the performance of gas-turbine, i.e. The higher AH in combustion air lowers NOx emissions by reducing peak flame temperature and enhances combustion efficiency, resulting in lower CO emissions in gas turbines. The sustained higher AH level for keeping the emissions in gas turbines at a lower level can contribute to environmental goals by lowering harmful pollutants like NOx and CO. The average and standard deviation of AH are $$0.72$$ and $$0.15$$, respectively. The coefficient of skewness is $$-0.54$$ indicates a negative skewed. The non-normality of the data is further confirmed by the Anderson-Darling $$({\rm A}=71.92$$ and $$p-value=0.000)$$ and Jarque-Bera Test $$(JB= 429.13, df = 2, p-value = 0.000)$$ (Fig. 1).

**Figure 2:**
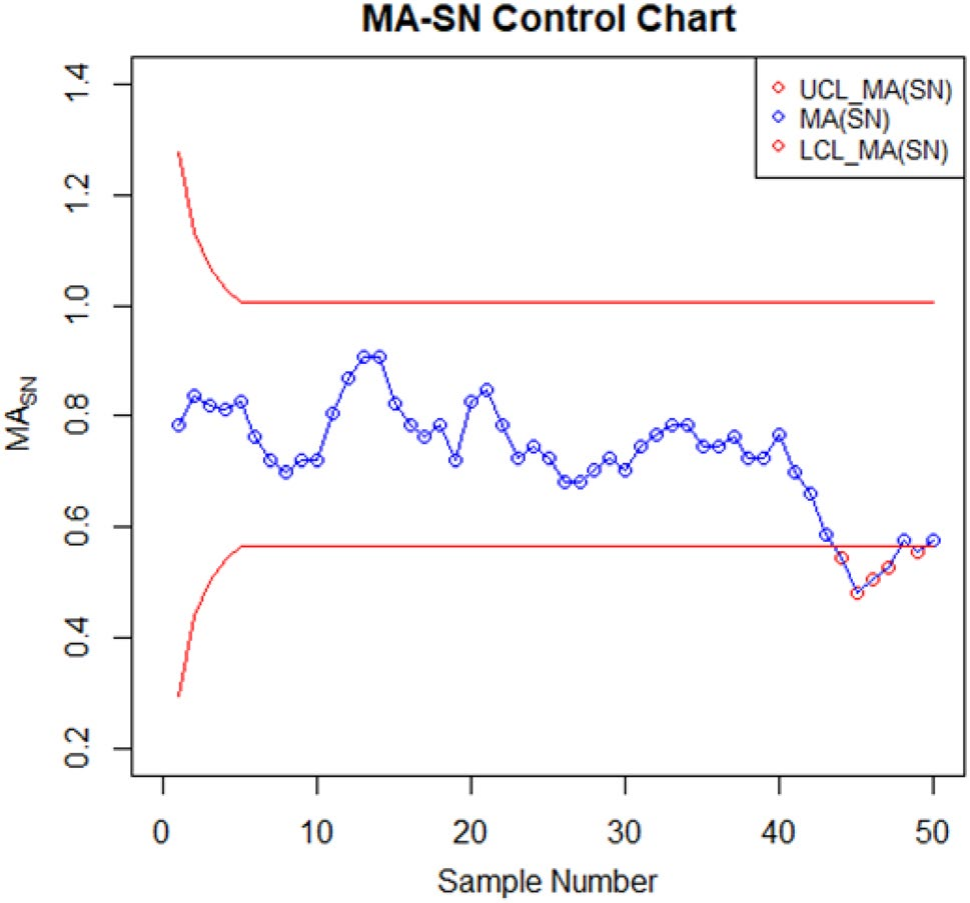
Nonparametric MA sign control chart of AH data.

**Figure 3:**
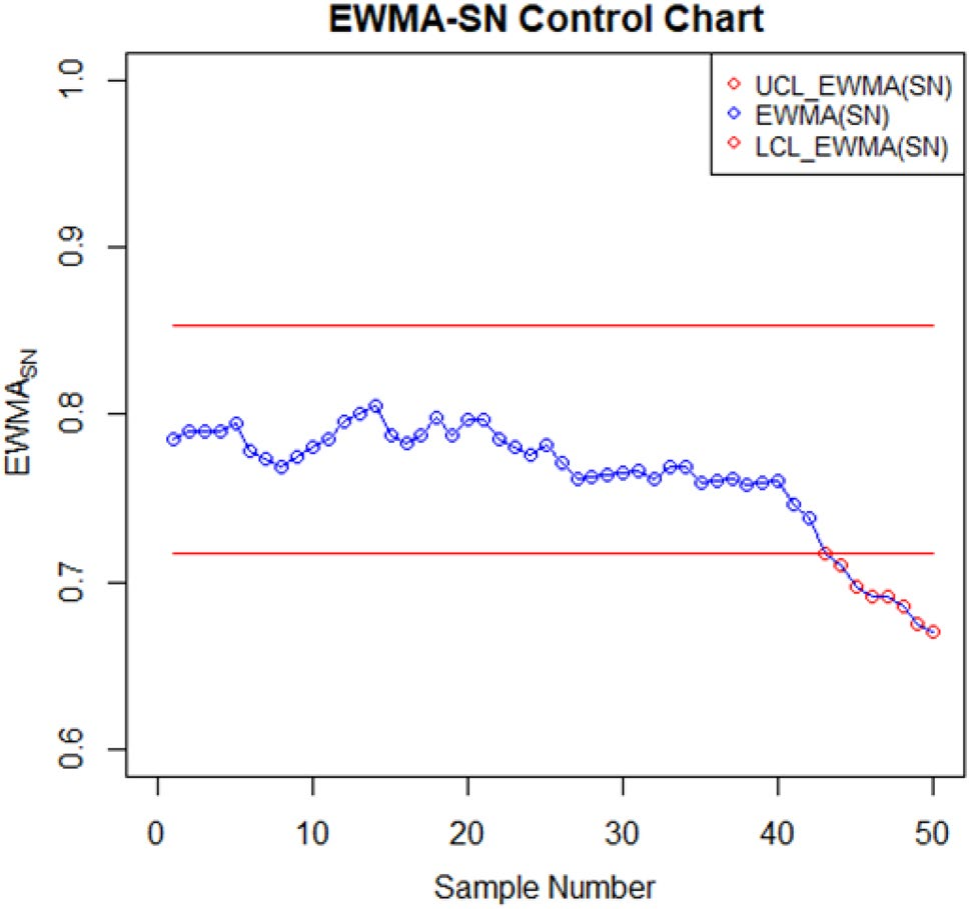
Nonparametric EWMA sign control chart of AH data.

**Figure 4:**
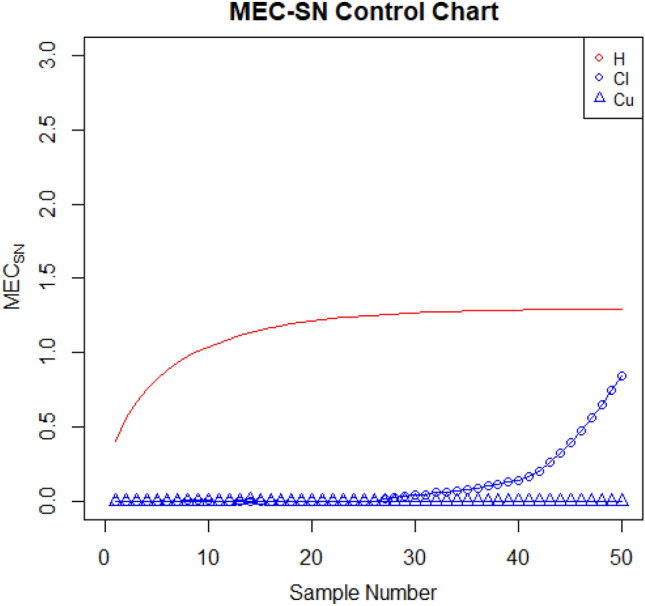
Nonparametric mixed EWMA-CUSUM sign control chart of AH data.

**Figure 5:**
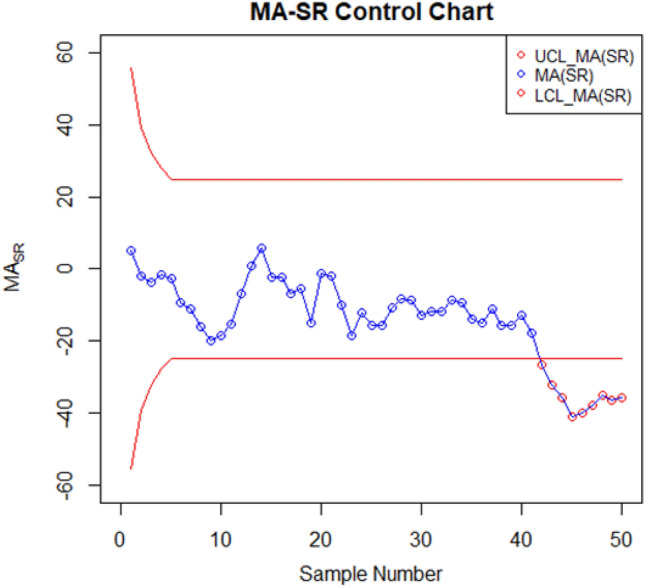
Nonparametric MA signed-rank control chart of AH data.

**Figure 6:**
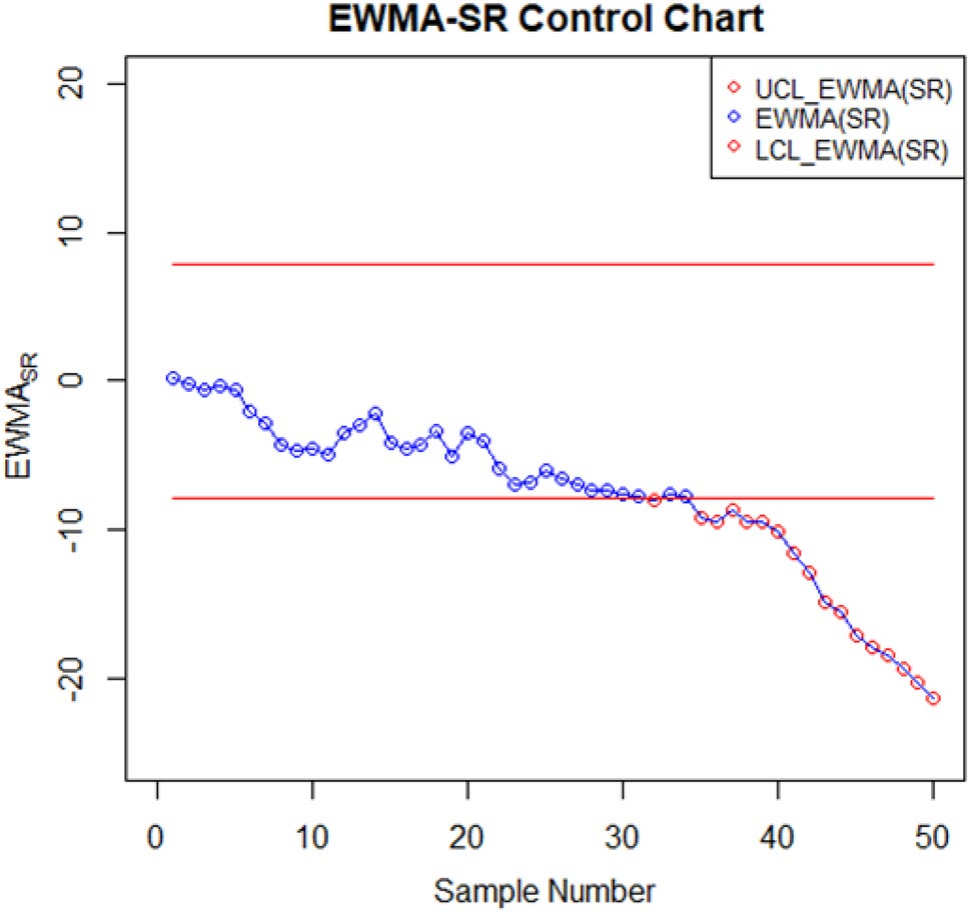
Nonparametric EWMA signed-rank control chart of AH data.

**Figure 7:**
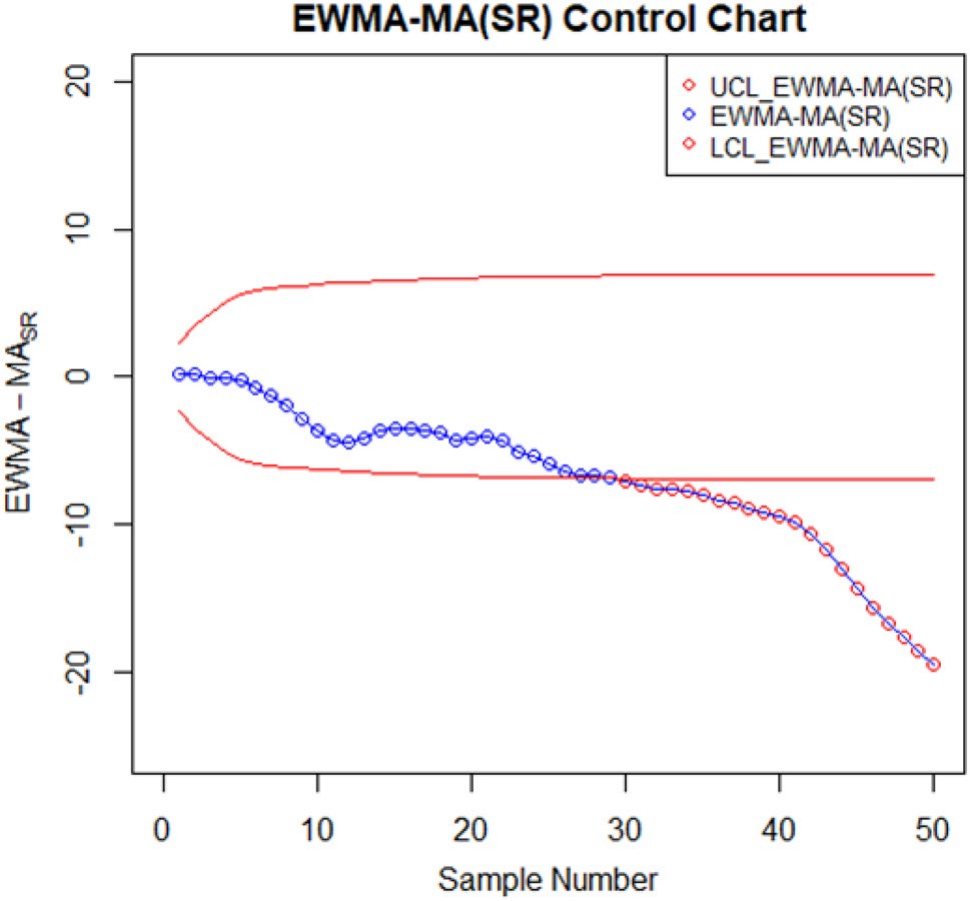
Distribution-free mixed EWMA-MA signed-rank control chart of AH data.

For setting up the control charts, 50 samples each consisting of 10 data points from the AH dataset are taken. The first 20 samples are considered to be IC, with a median value of $70.952$. To examine shift detection ability in the location parameter, we intentionally introduce a downward mean shift of $0.25\sigma$ in AH, and then subsequent 30 samples are generated under this shifted process. The proposed as well as the existing control charts are computed under a fixed ${ARL}_{0}\cong 370$. The MA-SN and MA-SR are constructed with parameters $w=5$ and $k= 3.095$ and $2.834$, respectively. Similarly, we use $\lambda =0.05$ and $k= 2.675$ and $2.481$ to setup the EWMA-SN and EWMA-SR control charts, respectively. The MEC-SN control chart is established with $\lambda =0.05$,$k=0.5$ and $h=51.28$. The EWMA-MA signed-rank control chart is computed with parameters $w=5$, $\lambda =0.05$, and $k=$ 2.304. Figures 2, 3, 4, 5, 6 and 7 illustrate the plotted monitoring statistics for the control charts against their corresponding control limits. The MA-SN chart from Fig. 2 triggers the first OOC signal at sample number 44, whereas the EWMA-SN chart, depicted in Fig. 3, is at sample number 43. The MEC-SN chart, shown in Fig. 4, declares the process as IC and does not produce an OOC signal. The MA-SR chart in Fig. 5 prompts the first OOC signal at sample number 42, while the EWMA-SR chart from Fig. 6 detects the initial OOC signal at sample number 32. Notably, in Fig. 7 the earliest OOC signal is detected by the EWMA-MA signed-rank control chart at sample number 30. These results further confirmed the superiority of the proposed control chart over its competitors, in line with the comparative run-length profiles.

## Summary and conclusion

In circumstances where the underlying distribution of a quality characteristic being monitored is unknown, nonparametric control charts offer a reliable and highly effective mechanism for monitoring a process. This study presented the distribution-free mixed EWMA-MA control chart, which is based on the signed-rank statistic for efficient detection of shifts in the process location. The run-length profile of the proposal is studied and compared with several competing control charts using extensive Monte Carlo simulations under a variety of symmetrical process distributions. Based on the obtained results, it is found that the proposed chart is more effective not only for detecting a specified shift in the process location but also in its overall ability to detect a range of shifts. In addition, a real-life example is provided to further validate the proposed chart's practicability and effectiveness in identifying process shifts in comparison to other competing control charts. The effectiveness of the proposed charting structure can be further explored for monitoring the process dispersion and joint monitoring of location and dispersion parameters. Moreover, a comprehensive investigation can be carried out to find the optimal values of the smoothing parameter and span for various shifts of interest.

## Data Availability

The data used in the paper was taken from Türkiye^58^.

## Abbreviations

EWMA: Exponentially weighted moving average
MA: Moving average
CUSUM: Cumulative sum
MEC: Mix EWMA-CUSUM
DEWMA: Double EWMA
GEWMA: Generally weighted moving average
DMA: Double moving average
AH: Ambient humidity
AT: Ambient temperature
AP: Ambient pressure
CO: Carbon monoxide
NOx: Nitrogen oxide
${\text{ARL}}$: Average run length
${\text{SDRL}}$: Standard deviation of run length
${\text{MRL}}$: Median run length
${\text{AEQL}}$: Average extra quadratic loss
${\text{RMI}}$: Relative mean index
$${\text{CL}}$$: Center line
$${\text{IC}}$$: In-control
${\text{OOC}}$: Out of control
${\text{LCL}}$: Lower control limit
${\text{UCL}}$: Upper control limit
${\text{SR}}$: Signed rank statistic
$${\text{SN}}$$: Sign statistic
